# Emotional Content Enhances Attentional Tracking of Conversations through Context-Sensitive Modulation of Auditory–Language Networks

**DOI:** 10.1523/JNEUROSCI.0506-26.2026

**Published:** 2026-10-26

**Authors:** Pyry Heikkinen, Santeri Lepistö, Patrik Wikman, Emmi Koskinen, Anssi Peräkylä, Niklas Ravaja, Ville Johannes Harjunen

**Affiliations:** ^1^Department of Psychology, Faculty of Medicine, University of Helsinki, Helsinki 00014, Finland; ^2^Finnish Centre of Excellence in Music, Mind, Body, and Brain, Cognitive Brain Research Unit, Department of Psychology, Faculty of Medicine, University of Helsinki, Helsinki 00014, Finland; ^3^Department of Neuroscience, Georgetown University, Washington, DC 20057; ^4^Advanced Magnetic Imaging Centre, Aalto NeuroImaging, Aalto University, Espoo 02150, Finland; ^5^Faculty of Social Sciences, University of Helsinki, Helsinki 00014, Finland

**Keywords:** attention, emotion, magnetoenchephalography, neural speech tracking, temporal response function

## Abstract

Engagement in conversation often requires continuous monitoring of relevant speech in a continuously evolving auditory environment. Emotional salience of semantic content may influence this process, but its role in attentional tracking of continuous speech remains unclear. We address this question using an audiovisual “cocktail-party” paradigm combined with source-localized magnetoencephalography and encoding–decoding analyses. Participants (18 females, 14 males) selectively attended to one dialogue while ignoring a concurrent distractor dialogue, with both emotional salience and semantic coherence of the conversations manipulated independently. Decoding analyses revealed that negatively valenced, high-arousal, and socially relevant semantic content strengthened neural speech tracking, but only for attended dialogues. This effect depended on semantic structure, emerging earlier in semantically coherent than incoherent dialogues. Encoding analyses further showed latency- and region-specific effects across temporal, parietal, and frontal cortical regions. Emotional salience and coherence jointly modulated midlatency (100–250 ms) responses, whereas late-latency (270–450 ms) responses were driven primarily by emotional salience. These findings demonstrate that emotional meaning enhances neural prioritization of attended speech and semantic coherence shapes the temporal dynamics of this enhancement. Together, these results indicate context-sensitive emotional modulation of auditory–language processing during selective listening of continuous speech.

## Significance Statement

Understanding how the brain prioritizes and selectively processes information in naturalistic, continuously evolving audiovisual scenes such as conversations is a central challenge in neuroscience. Here, we show that conversations conveying negatively valenced, high-arousal, and socially relevant content enhance neural tracking of attended speech in a noisy cocktail-party setting. This enhanced tracking depends on the semantic structure of the conversation, emerging earlier for coherent dialogues that are easy to follow. Furthermore, we show that emotional content strengthens mechanisms related to speech comprehension and sharpens segregation of relevant from irrelevant talk. These findings demonstrate that emotional meaning and semantic continuity jointly shape cortical processing of continuous speech, providing new insight into how the brain selects and processes meaningful information during a conversation.

## Introduction

When engaging in a conversation in noisy settings, attention plays a critical role in determining how speech is processed and represented in the brain. Emotional content, such as highly arousing and negative information with social significance, may bias this process by enhancing sensory processing of stimulus characteristics (Lang et al., 1998; Sander et al., 2003; Yiend, 2010; Fischer et al., 2025). However, it remains unclear whether emotional semantic content of speech modulates attentional selection by influencing neural tracking of naturalistic conversation.

At the neural level, much of what we know about emotional speech and language processing comes from studies examining responses to emotional words and prosody. These local emotional cues influence multiple processing stages, from early sensory encoding (e.g., N1) and salience detection (e.g., P2) to later evaluative stages (e.g., N400; Schirmer and Kotz, 2006; Kotz and Paulmann, 2011; Pinheiro, 2025). Their processing is further shaped by linguistic context, with emotionally congruent contexts facilitating emotional word processing through semantic predictability (van Berkum et al., 2003; Nieuwland and Van Berkum, 2006; Bayer et al., 2010; Fields and Kuperberg, 2012; Martín-Loeches et al., 2012; Ding et al., 2015, 2016; Zhang et al., 2021).

In everyday communication, emotional salience unfolds across continuous talk rather than isolated utterances (Mar, 2011; Hoemann et al., 2025). In such settings, the brain integrates information at multiple representational levels, from rapid acoustic fluctuations to slower semantic and contextual structure (Zion Golumbic et al., 2012, 2013; Brodbeck et al., 2022). This integration depends on coordination between auditory, language, and higher-order cognitive networks linking sensory input with semantic representations and contextual knowledge (Ji et al., 2019; Fedorenko et al., 2024; Yamashita et al., 2025). Neural responses to continuous speech reflect this organization, progressing from early spectrotemporal encoding to later lexical–semantic integration and contextual predictability (Zion Golumbic et al., 2013; Broderick et al., 2018).

In environments where relevant speech must be segregated from background noise, attention enhances neural tracking of the speech stream (Ding and Simon, 2012; Zion Golumbic et al., 2013; Fiedler et al., 2019; O’Sullivan et al., 2019). Segregating competing speech streams relies on integrating information across multiple timescales to support processing of the task-relevant stream (Power et al., 2012; Brodbeck et al., 2022; Teoh et al., 2022). Recent work shows that neural tracking of conversation fluctuates across turns and engages a distributed cortical network supporting auditory, linguistic, and social-cognitive processing (Wikman et al., 2021, 2024). Together, these findings raise the possibility that emotional meaning may influence neural tracking of conversational speech by interacting with distributed cortical systems that track the unfolding semantic context.

The present study examined whether emotional salience enhances neural tracking of conversational speech during selective listening. During magnetoencephalography (MEG) recording, participants listened to target dialogues while ignoring a competing speech stream in an audiovisual cocktail-party paradigm. We manipulated both the emotional salience of dialogue content and the semantic coherence of dialogue turns. The emotional-content condition comprised negatively valenced, highly arousing, and socially relevant dialogues. This profile is common in natural communicative settings (e.g., gossip, rumors; Berger and Milkman, 2012; Feinberg et al., 2012; Robbins and Karan, 2020), and combinations of these features are shown to promote attentional prioritization in sensory processing (Astudillo et al., 2018; Schacht and Vrtička, 2018; Fischer et al., 2025).

To characterize neural speech tracking, we combined speech envelope reconstruction (SER) with temporal response function (TRF) analyses. SER reconstructs the acoustic speech envelope from multivariate neural activity, whereas TRFs model stimulus–response relationships across time lags to estimate how fluctuations in the speech envelope drive cortical activity (Crosse et al., 2016; Kaufman and Zion Golumbic, 2023).

We hypothesized that negatively valenced, highly arousing, and socially relevant conversational content would enhance neural tracking of attended conversational speech and that dialogue coherence would modulate the temporal dynamics of this emotional enhancement. Finally, we predicted that emotional salience and dialogue coherence would differentially modulate neural responses across response latencies and cortical regions involved in language comprehension.

## Materials and Methods

### Participants

Thirty-two healthy volunteers (18 women, 14 men, age range 20–50 years, *M* = 30.22, SD = 7.98) took part in the study. Participants, recruited through university mailing lists and campus outreach, were asked during recruitment to report any diagnosed neurological or psychiatric disorders or use of medications affecting the central nervous system, all of which constituted exclusion criteria. Participants were also required to have normal or corrected-to-normal vision and hearing. All participants provided written informed consent before the experiment and received a reward valued at ∼30 euros. The research protocol was approved by the University of Helsinki Research Ethics Committee of the Faculty of Medicine, and the study followed the ethical principles outlined in the World Medical Association's Declaration of Helsinki. One participant was excluded due to data-quality issues, leaving a final sample of *N* = 31. For this participant, envelope-tracking quality-control diagnostics indicated unusable model output: SER performance was at or near zero across the tested regularization values, and TRF estimates showed atypically low-amplitude, highly stereotyped waveform patterns across regions in all conditions.

### Sensitivity power analysis

Given the final sample size (*N* = 31), we conducted a simulation-based sensitivity analysis for the planned SER and TRF analyses. The analysis estimated the minimum magnitudes of the principal SER and TRF effects detectable with 80% power at *α* = 0.05. Simulations preserved the observed variances and shrinkage-estimated within-participant correlation structures while varying the target effect. For each candidate magnitude, 5,000 datasets with 31 participants were analyzed using the corresponding Type III repeated-measures ANOVA (rm-ANOVA). Minimum detectable effects were reported in the original measurement units and as generalized eta squared $\left(\eta_{G}^{2}\right)$. Analyses were conducted in R 4.6.1 using Superpower (Lakens and Caldwell, 2021). For SER, simulations reproduced the 2 × 2 × 2 Attention × Emotion × Coherence design, and for TRFs, they reproduced the Emotion × Coherence × ROI designs used for the respective latency windows. The 80% power minimum detectable effect size for both the SER Attention × Emotion interaction and the Attention × Emotion × Coherence interaction was $\eta_{G}^{2}=0.033$. For the analyzed midlatency TRF response, the minimum detectable effect size at 80% power was $\eta_{G}^{2}=0.0029$. For the late-latency response, the minimum detectable effect size at 80% power was $\eta_{G}^{2}=0.0109$.

### Experimental design

The participants completed an audiovisual “cocktail-party” experiment during MEG recording (Fig. 1). After a 4 min resting-state recording, participants performed two experimental runs consisting of 16 trials each. In each trial, two dialogues with six lines were presented simultaneously: an audiovisual target dialogue and an audio-only distractor dialogue, with audio presented from the same speakers concurrently. Participants were instructed to focus exclusively on the target dialogue. Interactants in the dialogues did not exhibit bodily expressions (e.g., facial expressions, hand gestures, or postural movement). Crucially, to focus the manipulation on semantic content, the speech was delivered with flat prosody, minimizing variation in stress, rhythm, and loudness.

**Figure 1.**
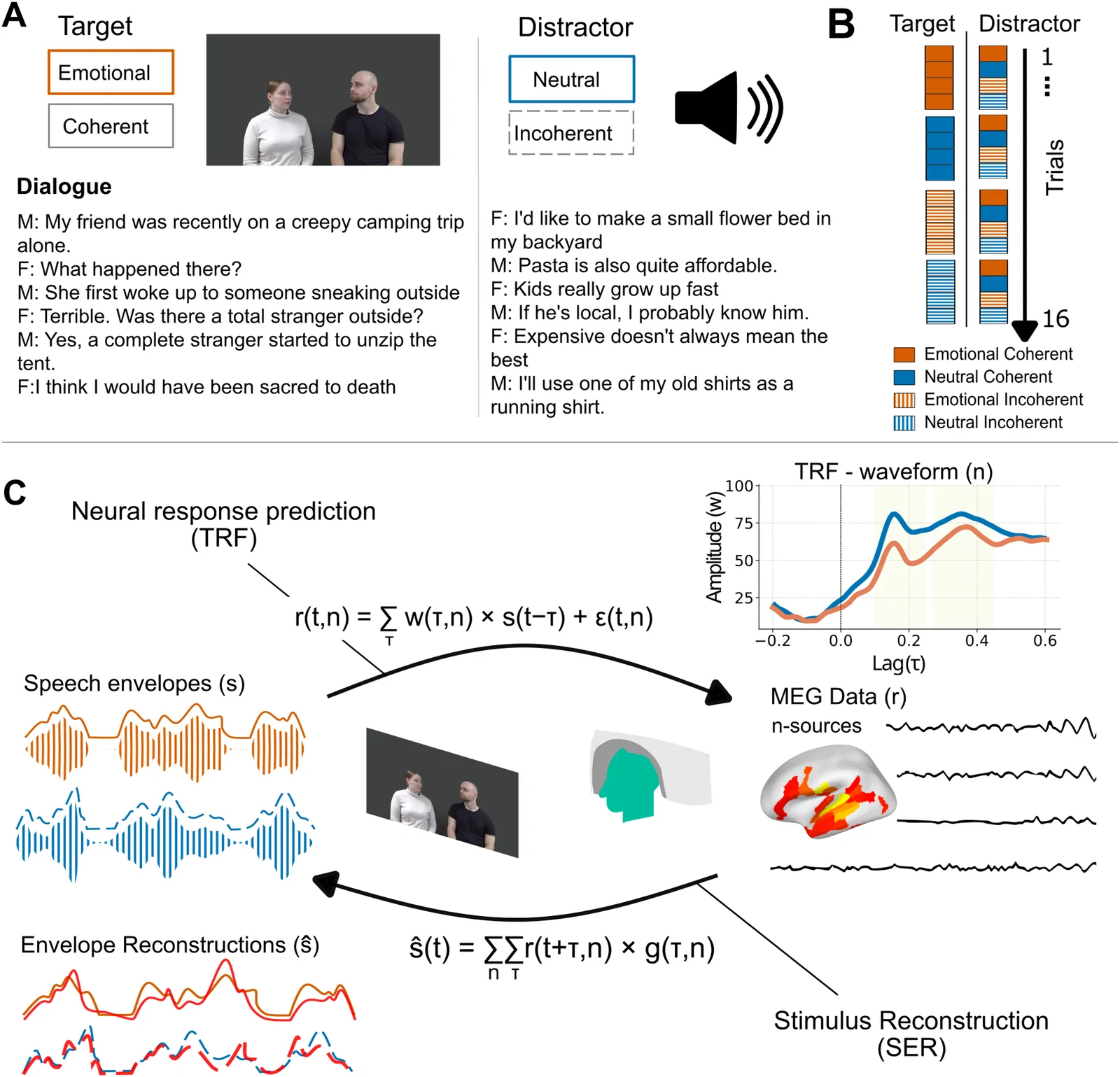
The experimental design and analysis framework. ***A***, Example stimulus materials. The left panel shows an example of an audiovisual target dialogue, including the video stimulus and transcript, illustrating an emotionally salient coherent dialogue. The right panel shows a corresponding audio-only distractor dialogue, exemplifying a neutral incoherent conversation. ***B***, Structure of a single trial, in which each target dialogue type was presented with each distractor type. Trials are shown grouped by target condition for illustration; the actual presentation order was pseudorandomized (see Materials and Methods). ***C***, Schematic overview of the modeling approaches. Speech envelopes of the target (orange) and distractor (blue) dialogues for each condition were used separately to predict how neural activity responded to changes in the speech envelope with TRFs. In the SER analysis, neural activity was used to reconstruct the corresponding speech envelopes. Note that presented reconstructions and TRF waveforms are for illustrative purposes only.

The experiment followed a 2 × 2 × 2 factorial design, manipulating attentional engagement (target vs distractor), and two dimensions of semantic content in both target and distractor dialogues: the emotional salience of the dialogues (emotional vs neutral) and turn-to-turn dialogue coherence (narratively coherent dialogue vs narratively incoherent dialogue). Emotional dialogues featured negatively valenced topics, whereas neutral dialogues described affectively nonsalient activities (details of the dialogue material are provided below, Dialogue set construction). Coherent dialogues maintained contextually related conversational turns, while incoherent dialogues were generated by mixing lines from different dialogues. Each trial within a run formed a different factorial pairing, resulting in 16 unique target–distractor combinations. For example, both runs included a trial in which an emotionally incoherent target dialogue was presented alongside a neutral coherent distractor dialogue. The trial order was pseudorandomized.

### Stimulus material

#### Dialogue set construction

The stimulus material comprised a set of 128 spoken dialogues developed specifically for the present study, with conversations varying in emotional salience (emotional vs neutral) and coherence (coherent vs incoherent). All dialogues were scripted by two research assistants and the last author. Each consisted of a six-line exchange of ∼30 s, with semantic content manipulated according to the intended experimental condition. For each participant, a subset of 32 target dialogues and 32 distractor dialogues was selected from this larger stimulus set. Each dialogue was recorded by two different male–female speaker pairs (Dyads A and B), yielding two versions of each dialogue with different speakers. The speaker pair was counterbalanced across participants: for half of the participants, Dyad A performed the target dialogues and Dyad B the distractor dialogues, whereas for the other half, this assignment was reversed. The gender of the active speaker also differed between the target and distractor dialogues.

The level of emotional salience was manipulated by constructing dialogues around two topic clusters that differed in their apparent emotional salience. Emotional dialogues were about negatively valenced, emotionally arousing events with high social relevance (e.g., tragic accident, fear- or disgust-related experience, physical conflict, mental health issue or illness, harassment, social exclusion), whereas neutral dialogues centered on neutral, everyday events with low emotional arousal and low social significance (e.g., grocery shopping, attending a lecture, cooking, working, talking about the weather, having lunch, drinking coffee). The emotional salience of the stimuli was validated in a separate sample (see below).

In each dialogue, information concerning the topic unfolded across the six conversational turns (i.e., each turn introduced new emotionally salient or neutral content). The level of semantic coherence was manipulated by varying the semantic relatedness of the interactants' utterances (turn-to-turn contextual continuity). In coherent dialogues, interactants responded appropriately to each other's speech, whereas in incoherent dialogues, the turns were not semantically connected. Incoherent dialogues were constructed by recombining utterances from different coherent dialogues to form new dialogues with unrelated successive turns (e.g., one interactant makes a comment about gardening, the other responds with an unrelated remark about shopping). The resulting coherence manipulation was independently validated using dialogue ratings and natural language processing-based measures of semantic dispersion (see below).

#### Independent dialogue validation study

To ensure the dialogue materials were in accordance with our experimental design, we conducted a separate dialogue validation study administered online using a custom jsPsych-based questionnaire (de Leeuw, 2015). The dialogue validation study utilized the full set of 128 dialogues, with each participant viewing and evaluating a subset of 16 dialogues comprising 4 dialogues from each experimental condition (emotional coherent, emotional incoherent, neutral coherent, and neutral incoherent). Participants were primarily recruited through university campus outreach. The validation study sample included 31 participants (14 females, 16 males, and 1 reporting “other/prefer not to disclose”), with ages ranging from 22 to 70 years (*M* = 32.71, SD = 8.68).

Participants rated each dialogue on five dimensions—arousal, valence, social relevance, visual imagery, and semantic coherence—using nine-point Likert-type scales. Arousal and valence were assessed using the Self-Assessment Manikin (SAM; Bradley and Lang, 1994). Social relevance was assessed with the question “To what extent did the dialogue concern people, relationships, or social interaction?” (1, not at all; 9, very much). Visual imagery was assessed with the question “How easy was it to imagine the events described in the dialogue?” (1, very difficult; 9, very easy). Finally, perceived coherence was assessed using the statement “Did the dialogue progress coherently” (1, strongly disagree; 9, strongly agree).

#### Lexical and semantic feature extraction

To examine if the dialogue categories differed on their linguistic and semantic properties, we extracted the lexical and semantic features of each dialogue. These variables were used first to examine any possible differences between the categories and then in follow-up analyses testing whether they accounted for observed effects in SER.

We used lexical-control variables derived from LASTU, a psycholinguistic search tool and lexical database for Finnish stimuli (Itkonen et al., 2024). The dialogue transcripts were lemmatized (returned to base form) with STANZA (Qi et al., 2020), and each word was matched to the corresponding LASTU entry. Dialogue-level lexical variables were then calculated by averaging token-level values across all matched lexical tokens within each dialogue.

Mean word frequency was calculated as the average log-transformed lemma frequency across matched tokens in the dialogue, with higher values indicating more frequent occurrence in the Finnish Internet Parsebank corpus (Luotolahti et al., 2015). Mean word length was calculated as the mean character count of words within a dialogue. Mean word ambiguity was calculated as the average LASTU lemma-ambiguity value, with higher values indicating words associated with more possible lexical or morphological interpretations. Mean morphological feature count was calculated as the average number of morphological feature tags assigned to the actual words in the dialogue, indexing the degree of morphological complexity of the dialogue content.

In addition to the above lexical features, dialogue-level narrative density and semantic spread were used to characterize the overall narrative structure of the dialogues. Narrative density indexed the extent to which a dialogue contained explicit event-structure cues, including references to actions or events, causal or temporal relations, goals or intentions, and human participants. Higher values therefore indicated dialogues with denser event-based and causal–temporal content.

Finally, semantic dispersion was computed using sentence embeddings generated with the Finnish Sentence-BERT paraphrase model (Kanerva et al., 2024). This model is a Finnish sentence transformer trained from FinBERT and produces sentence-level embeddings suitable for estimating semantic similarity between sentences (Reimers and Gurevych, 2019; Virtanen et al., 2019). Semantic dispersion was then calculated as the average cosine distance among the turn embeddings within a dialogue, providing a dialogue-level index of how semantically spread out the turns were.

### Stimulus presentation

The stimulus was presented with the Presentation software (Neurobehavioral Systems). The target dialogues were presented on a projected screen (67 × 38 cm) at a viewing distance of ∼130 cm (from the participant's eyes). The projector's resolution was 1,920 × 1,080 (full HD), with a refresh rate of 120 Hz. Before each target dialogue, a gray screen with the instruction “Pay attention to the conversation” was shown. Subsequently, target and distractor dialogues were presented line by line. After each line, a gray screen with a fixation cross was presented. For each line, the stimulus volume was set to a comfortable level based on the target, and the paired distractor was attenuated by 10 dB based on the root mean square of the amplitude, and each distractor line was set to begin 250 ms before the onset of the corresponding target line.

### Stimulus processing

The broadband amplitude envelope of each dialogue was obtained as the absolute value of the Hilbert transform. Audio envelopes were band-pass filtered (0.5–15 Hz, fourth-order Butterworth filter), and the sampling rate was matched to MEG data at 128 Hz. Finally, the envelopes were segmented into speech epochs for each line by detecting silent gaps of ≥1 s where the envelope remained below 1% of the maximum amplitude. As the distractor dialogue lines started 250 ms prior to the targets, the start of distractor epochs was cut to align with the onset of target lines.

### Behavioral measures

After each trial, participants completed two questionnaires with a VPixx response device. To evaluate whether participants attended the target dialogues, they answered four true–false statements about the content of the target dialogue. The statements were designed to measure participants' detection of central semantic information conveyed in the dialogues, such as recognizing mentioned people, places, objects, or actions (e.g., “The interactant asked whether the other person had an umbrella”; “The interactant is looking for a job”; “The interactant said that their lecturers change often”). Each true–false statement was constructed with its correct answer randomly set as either true or false. After responding, participants received feedback (e.g., “3 out of 4 correct”). The proportion of correct responses was used to quantify recall accuracy (number of correct responses divided by four). Second, participants rated how emotionally charged the dialogue was on a three-point scale (1, “Not at all”; 2, “Somewhat”; 3, “Highly”).

### MEG and MRI data acquisition

For source localization, structural magnetic resonance image (MRI) data were acquired using a 1.5 T Siemens Avanto scanner with a T1-weighted 3D magnetization-prepared rapid gradient-echo sequence (voxel size, 1 × 1 × 1 mm^3^). Brain activity during the experiment was recorded at 1 kHz with a 306-channel MEGIN Triux system (204 planar gradiometers, 102 magnetometers) at the BioMag Laboratory, Helsinki University Hospital. Horizontal and vertical electro-oculograms (EOG), as well as electrocardiograms (ECG), were recorded to detect eye movements and cardiac artifacts. The head positions of the participants were continuously measured using five head position indicator (HPI) coils placed around the scalp. To facilitate accurate coregistration between the MEG data and a structural MRI, 100 evenly divided scalp points were digitized (Polhemus).

### Data processing

#### MEG preprocessing

MEG signals were first preprocessed using MNE-Python 1.5 (Gramfort et al., 2013). Raw recordings were denoised using MNE MaxFilter (Taulu and Simola, 2006) with temporal signal space separation (tSSS; 20 s sliding window). The head position of each participant was spatially aligned to the start of Run 1, with movement compensation based on the recorded continuous HPI coil information. Noisy and flat channels were automatically marked, and the data were resampled to 128 Hz. Data were high-pass filtered at 0.5 Hz and low-pass filtered at 15 Hz using zero-phase FIR filters. The 0.5–15 Hz passband was chosen to reduce slow drifts while retaining low-frequency neural activity and speech envelope modulations relevant for continuous speech tracking, including delta- and theta-range fluctuations and adjacent syllabic/onset-related structure. Similar low-frequency passbands extending to 15 Hz have been used in TRF and stimulus-reconstruction analyses of continuous speech (Di Liberto et al., 2015; Crosse et al., 2016).

Bad channel selection was manually confirmed, and channels were interpolated with spherical-spline interpolation. Next, ocular and cardiac artifacts were removed via the MNE FastICA algorithm by flagging components correlated with the EOG and ECG, respectively, and manually confirming the ICA exclusion. Next, MEG data were epoched from each dialogue line onset to offset for both target and distractor dialogues separately. Finally, the data were grouped based on emotional salience and semantic coherence, yielding separate neural data and audio envelope epochs for each Attention–Emotional Salience–Coherence combination.

#### Distributed source localization

To estimate the cortical generators of the measured MEG signal, distributed minimum-norm source reconstruction (Hämäläinen and Ilmoniemi, 1994; Gramfort et al., 2014) was performed with each participant's FreeSurfer reconstructed surfaces. The surfaces were coordinate aligned to MEG digitization points, and a single-shell boundary element head model (conductivity 0.3 S/m) was computed from the inner skull surface. The cortical source spaces were defined at oct6 resolution (∼5 mm spacing) on the white matter surface. Noise covariance matrices were estimated from 4-min-long empty-room recordings. Minimum-norm inverse operators were built (depth weighting, 0.2; SNR, 1/9) with dipole orientation constrained to the cortical surface normal using a loose orientation constraint (0.2). MEG data were projected to source space separately for each MEG epoch, yielding one cortical source current estimate time-locked to each stimulus combination.

### Encoding and decoding models

Encoding and decoding modeling were used to examine how distributed cortical activity tracked the auditory envelopes of the presented dialogues, utilizing TRFs and SER (Lalor and Foxe, 2010; Crosse et al., 2016). By using SER, it is possible to assess the fidelity with which the cortical activity follows the speech signal, whereas TRFs characterize the temporospatial dynamics of stimulus–response relationship (Crosse et al., 2016). The temporal lags considered in the models between speech signal and brain activity were restricted to be between −200 and 600 ms. For both approaches, the *receptive field estimation* function in MNE-Python was used, with the default lagged ridge-regression approach, with fourfold cross-validation (regularization parameters tested between 1 and 1 × 10^6^; SI Fig. S1). Separate models were fit for each Attention–Emotional Salience–Dialogue coherence combination. Model performance was quantified as the mean Pearson’s correlation between model predictions and held-out test data across cross-validation folds. The final selection was based on the highest overall correlation value, averaged across conditions. For the encoding models, a regularization value of *λ* = 10^4^ provided the best performance and was therefore used in the final analyses. The same *λ* was used for SER to keep regularization matched across encoding and decoding models.

#### SER

To reduce computational strain, the neural input data were first condensed to anatomically functionally defined cortical areas, based on the human connectome project's multimodal parcellation (Glasser et al., 2016) by calculating the average time course within each cortical area (with sign-flip of activations in the nondominant orientations with >90° deflection). Reconstruction accuracy was quantified as Pearson's product–moment correlation coefficient of the reconstructed and true envelopes across cross-validation folds.

To examine how speech tracking changed across dialogue turns, we used the envelope reconstructions of the held-out data from the overall cross-validated SER analysis. The reconstructed and actual envelopes were segmented according to the dialogue-turn boundaries, and turn-wise SER accuracy was calculated as the Pearson’s correlation between the reconstructed and actual envelopes within each turn. Correlation coefficients were Fisher *z*-transformed before statistical analysis. Overall SER accuracy and turn-wise SER trajectories were analyzed separately.

#### TRFs

TRFs were used to model how changes in the speech envelope drove cortical responses over time with the envelope as the input and the neural source activity at each vertex as the response. Regression coefficients were averaged across folds to yield source-by-time TRFs for each subject and condition and were subsequently averaged based on the Glasser cortical parcellation. From this partition, the auditory, language, and default-mode–related regions located in the lateral temporal, parietal, and frontal areas were chosen for further analysis. As the grand-averaged responses displayed a distinct two-peak response pattern, the data were analyzed in two separate time windows of interest. For the earlier window, a local positive peak within 100–250 ms was identified for each network and condition separately. The mean amplitude within a 50 ms window around this peak was computed, capturing the midlatency response while accommodating latency differences across cortical regions. The later activation was quantified as the overall mean across 270–450 ms time window, due to less pronounced curvature in the source waveforms.

### Statistical analyses

All statistical analyses were performed in R (version 4.5.1). Statistical significance was assessed at *α* = 0.05, and effect sizes are reported as $\eta_{G}^{2}$. Post hoc comparisons were conducted when applicable.

#### Dialogue-level validation

To validate the differences between stimulus categories and characterize possible differences between them in lexical or semantic features, we conducted dialogue-level control analyses comparing factors of emotional content (emotional vs neutral) and coherence (coherent vs incoherent). The analyses were conducted at the dialogue level, with each of the 128 dialogues acting as one observation. The used questionnaire-derived variables were dialogue-level mean ratings of arousal, valence, social relevance, visual imagery, and perceived coherence, and the used lexical variables were mean log word frequency, mean word length, mean word ambiguity, and mean morphological feature count. Finally, we used semantic variables of narrative density and embedding-based semantic dispersion across dialogue turns. Continuous outcome variables were standardized before modeling. Type III tests were used for the analyses, with false discovery rate (FDR) correction applied across the set of tests (Benjamini and Hochberg, 1995).

#### Behavioral analysis

For the MEG study participants, emotional intensity ratings were analyzed using generalized estimating equations (GEE) with an ordinal link function. Recall accuracy and SER accuracy were analyzed using rm-ANOVAs with within-subject factors of Attention (attended, distractor), Emotional Salience (emotional, neutral), and Dialogue Coherence (coherent, incoherent).

#### SER and TRF analysis

To assess temporal changes in neural tracking across conversational turns, SER trajectories were analyzed using rm-ANOVAs with Turn included as an additional within-subject factor. When the assumption of sphericity was violated, Greenhouse–Geisser corrections were applied. Multiple comparisons were corrected using the Holm correction.

To control for possible effects of lexical or semantic features in speech tracking, we tested if any of the ancillary lexical or discourse-level variables accounted for the observed effects in target SER. For this, a baseline categorical model predicting dialogue-level target SER from emotional-content condition, coherence, and their interaction was compared with models additionally including either lexical or semantic-level predictors. The model fits were evaluated using Akaike information criterion (AIC) and Bayesian information criterion (BIC; Akaike, 1974; Schwarz, 1978).

Next, we examined whether the independently rated affective–social dimensions provided a stronger continuous account of target SER than the categorical manipulation. These models tested arousal, valence, social relevance, and their interactions and were compared with the planned model with categorical factors of emotion and coherence. The model fits were evaluated using AIC and BIC. Together, these follow-up analyses test whether the categorical SER effect persists after accounting for measured stimulus differences and clarify the relation of SER to the dialogues' affective–social profile.

Finally, TRF amplitudes were averaged within predefined latency windows (midlatency, 100–250 ms; late-latency, 270–450 ms) and analyzed using rm-ANOVAs with factors Emotional Salience, Dialogue Coherence, Hemisphere, and Region of Interest (ROI). Multiple comparisons in topographical analyses were controlled using FDR correction.

## Results

The results are presented in four stages. We first report the dialogue-level validation and lexical–semantic control analyses, followed by behavioral results from the MEG study. We then examine the effects of emotional salience and dialogue coherence on neural speech tracking. Finally, TRF analyses characterize the temporal and cortical organization of emotional enhancement in neural speech tracking.

### Dialogue validation and lexical–semantic control analyses

Independent ratings confirmed the intended differences in both emotional-content and coherent categories (SI Fig. S2*A*). Emotional-content dialogues were rated as more arousing, (*F*_(1,124)_ = 1,122.70, *p* < 0.001), lower in valence (*F*_(1,124)_ = 991.47, *p* < 0.001), and more socially relevant (*F*_(1,124)_ = 106.99, *p* < 0.001) than neutral dialogues. Coherent dialogues were rated as more coherent than incoherent dialogues (*F*_(1,124)_ = 2,738.30, *p* < 0.001). Perceived coherence also showed an Emotion × Coherence interaction (*F*_(1,124)_ = 17.33, *p* < 0.001), reflecting a somewhat smaller coherent–incoherent difference for emotional-content dialogues.

The categories also differed in several lexical and semantic dimensions (SI Fig. S2*B,C*). Coherent dialogues were easier to imagine than incoherent dialogues (*F*_(1,124)_ = 132.51, *p* < 0.001), and emotional-content dialogues were slightly more imageable than neutral dialogues (*F*_(1,124)_ = 5.70, *p* = 0.041). Semantic dispersion was higher for incoherent than coherent dialogues (*F*_(1,124)_ = 199.52, *p* < 0.001) and also varied with emotional content (*F*_(1,124)_ = 41.71, *p* < 0.001), with an Emotion × Coherence interaction (*F*_(1,124)_ = 22.78, *p* < 0.001). Narrative density was higher in emotional-content dialogues (*F*_(1,124)_ = 5.23, *p* = 0.049). Neutral dialogues contained higher-frequency (*F*_(1,124)_ = 15.65, *p* < 0.001) and longer words (*F*_(1,124)_ = 8.04, *p* = 0.013); word ambiguity and morphological feature count showed no reliable effects. As several ancillary lexical and discourse-level properties differed between categories, we ran follow-up analyses testing whether these differences accounted for the effects observed in neural data.

### Behavioral results

As part of the main MEG experiment, each dialogue was followed by two behavioral measures: a rating of perceived emotional load and true–false questions assessing recall accuracy of the dialogue content.

Emotional dialogues (*M* = 2.40, SD = 0.69) were perceived as more affectively charged when compared with neutral ones (*M* = 1.06, SD = 0.24, ordinal GEE estimate = 14.14, SE = 0.14, *p* < 0.001). This indicated that participants were sensitive to the emotionality embedded in the conversation content. Neither dialogue coherence (ordinal GEE estimate = 0.08, *SE* = 0.04, *p* = 0.06) nor the Emotion × Coherence interaction (ordinal GEE estimate = −0.003, SE = 0.04, *p* = 0.93) significantly affected perceived emotional load.

Recall accuracy for the coherent dialogue content was higher (*M* = 0.89, SD = 0.18) than for incoherent ones (*M* = 0.80, SD = 0.21), implying that semantic continuity had facilitatory effect on memory performance (*F*_(1,30)_ = 53.73, *p* < 0.001, $\eta_{G}^{2}=0.199$). Emotional salience had no significant effect on recall accuracy (*F*_(1,30)_ = 0.81, *p* = 0.376, $\eta_{G}^{2}=0.004$), and there was no Emotion × Coherence interaction (*F*_(1,30)_ = 0.08, *p* = 0.779, $\eta_{G}^{2}<0.001$).

### Emotional salience enhances neural tracking of attended speech

SER showed better accuracy for attended, compared with distractor speech, demonstrating a robust attentional enhancement in neural speech tracking, rm-ANOVA (*F*_(1,30)_ = 408.79, *p* < 0.001, $\eta_{G}^{2}=0.56$; Table 1). SER accuracy was also higher for emotional than for neutral dialogues (*F*_(1,30)_ = 26.66, *p* < 0.001, $\eta_{G}^{2}=0.06$), whereas coherence had no impact on overall tracking accuracy (*p* = 0.83). Importantly, the influence of emotional salience depended on attentional engagement: emotional content enhanced SER only for attended speech, regardless of dialogue coherence (Fig. 2*A*; Attention × Emotion × Coherence interaction: *F*_(1,30)_ = 6.17, *p* = 0.019, $\eta_{G}^{2}=0.02$; SI Table S1).

**Figure 2.**
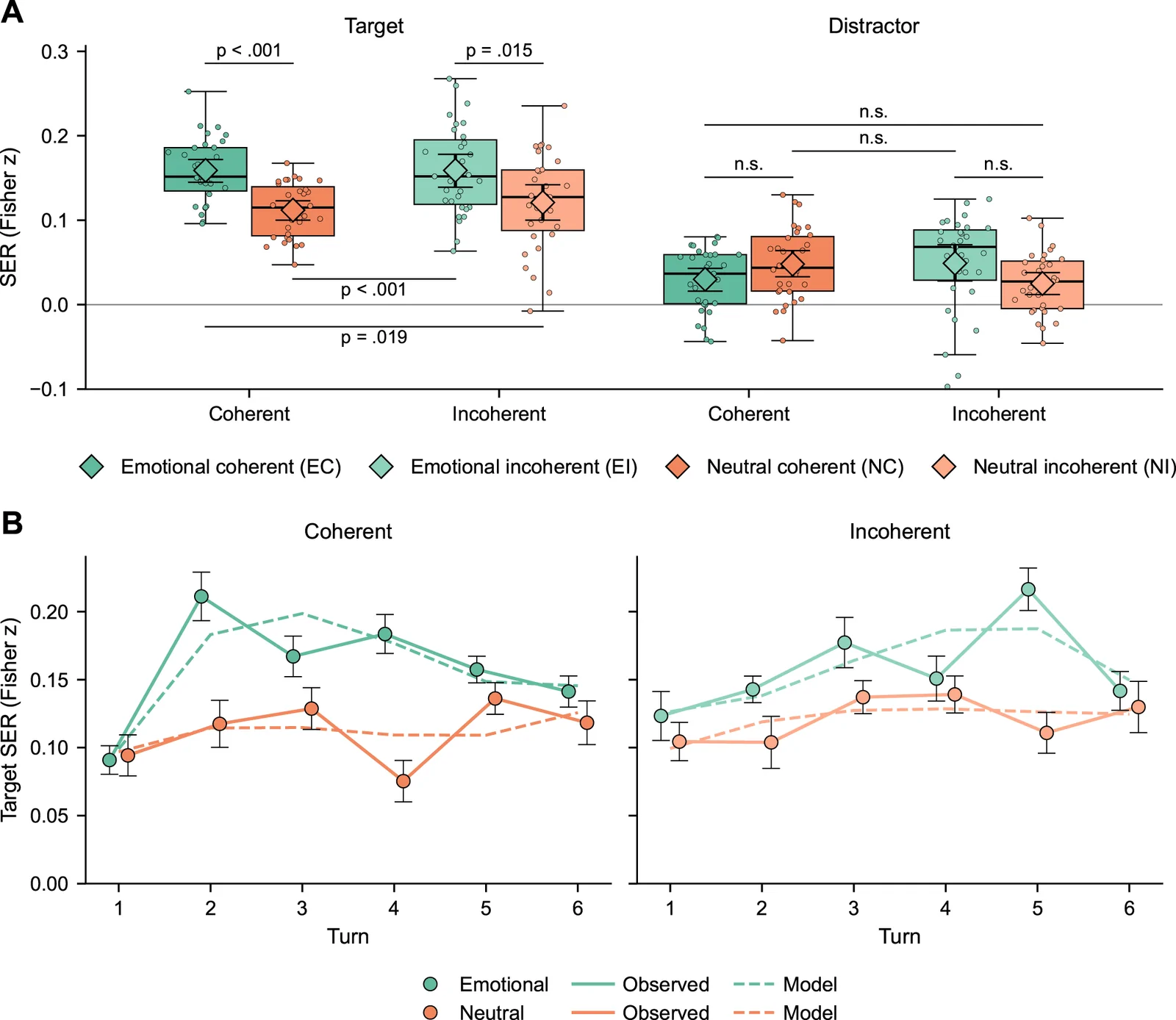
Emotional salience enhances and temporally modulates neural speech tracking. ***A***, Mean SER accuracy (Fisher's *z*-transformed) for target and distractor speech as a function of emotional salience and dialogue coherence. Boxplots show empirical distributions, with individual observations overlaid. Diamonds indicate estimated marginal means with 95% confidence intervals. Horizontal bars indicate Holm-adjusted pairwise comparisons; exact *p* values are shown for significant contrasts and n.s. for the corresponding nonsignificant distractor contrasts. ***B***, Turn-by-turn trajectories of SER accuracy for attended speech. Points and solid lines show observed means with standard error bars. Dashed lines indicate cubic model predictions, illustrating an earlier peak for coherent emotional dialogues followed by attenuation and a later, more sustained increase for incoherent emotional dialogues.

**Table 1.** rm-ANOVA for overall SER accuracy

Effect	df	*F*	$\eta_{G}^{2}$	*p*
Attention	1, 30	408.79	0.56	<0.001
Emotion	1, 30	26.66	0.06	<0.001
Coherence	1, 30	0.05	0.00	0.831
Attention × Emotion	1, 30	13.11	0.05	0.001
Attention × Coherence	1, 30	0.36	0.00	0.555
Emotion × Coherence	1, 30	2.31	0.01	0.139
Attention × Emotion × Coherence	1, 30	6.17	0.02	0.019

Type III rm-ANOVA testing the within-participant effects of Attention (attended, distractor), Emotional Salience (emotional, neutral), and Dialogue Coherence (coherent, incoherent) on overall SER accuracy. Generalized eta squared $\left(\eta_{G}^{2}\right)$ is reported as the effect size. Exact *p* values are reported unless *p* < 0.001.

We next tested whether the differences in lexical and semantic properties of the dialogue categories accounted for the emotional-content effect on target SER. The categorical Emotion × Coherence model provided the best fit with the data (AIC = −434.08, BIC = −419.82). Adding lexical controls did not improve model fit (AIC = −428.97, BIC = −403.30), nor did adding semantic control variables (AIC = −427.45, BIC = −401.78). In both control models, the emotional-content effect remained stable (SI Table S2).

Because emotional-content dialogues were found to be lower in valence and higher in arousal and social relevance, we next asked whether target SER was primarily associated with any one of these dimensions or with their combination and whether the dimensional account outperformed the planned categorical Emotion × Coherence model. The continuous model showed a significant arousal × valence × social relevance interaction (*b* = 0.0155, SE = 0.0076, *t* = 2.03, *p* = 0.044; SI Table S2). However, it provided a less parsimonious account than the categorical emotional-content model (AIC = −426.95, BIC = −398.43 compared with AIC = −434.08, BIC = −419.82). Thus, we retain the categorical model as our main focus when interpreting the findings.

### Emotional salience modulates temporal dynamics of speech tracking

As neural tracking of audiovisual conversation stimuli has been shown to vary according to the unfolding dialogue (Wikman et al., 2021, 2024), we further examined how neural tracking unfolds across conversational turns. Emotional salience affected the temporal evolution of SER and was modulated by dialogue coherence (rm-ANOVA: *F*_(4.23,126.98)_ = 4.58, *p* = 0.001, $\eta_{G}^{2}=0.03$; Table 2). Specifically, Emotional Salience selectively increased the parabolic arc of the SER trajectory in coherent dialogues, whereas dialogue coherence modulated the timing of emotional effects, with coherent emotional dialogues showing an earlier peak, compared with a more sustained increase in incoherent dialogues (Fig. 2*B*; SI Table S1).

**Table 2.** Turn-wise rm-ANOVA for attended SER accuracy

Effect	df	*F*	$\eta_{G}^{2}$	*p*
Emotion	1.00, 30.00	31.04	0.06	<0.001
Coherence	1.00, 30.00	0.21	0.00	0.652
Turn	4.02, 120.56	8.47	0.04	<0.001
Emotion × Coherence	1.00, 30.00	0.24	0.00	0.627
Emotion × Turn	4.32, 129.71	3.18	0.02	0.013
Coherence × Turn	4.33, 129.92	3.33	0.02	0.010
Emotion × Coherence × Turn	4.23, 126.98	4.58	0.03	0.001

Type III rm-ANOVA testing the within-participant effects of Emotional Salience, Dialogue Coherence, and conversational Turn on SER accuracy for attended speech. Greenhouse–Geisser-corrected degrees of freedom are reported where the assumption of sphericity was violated. Generalized eta squared $\left(\eta_{G}^{2}\right)$ is reported as the effect size. Exact *p* values are reported unless *p* < 0.001.

### Emotional modulation of cortical responses to continuous speech

To examine how emotional salience affects the temporal and topographical organization of neural speech processing, we analyzed TRFs for emotionally salient and neutral dialogues in both coherent and incoherent conditions. Grand-averaged TRFs exhibited a distinct two-peak structure, with the strongest activations in temporal–frontal regions (Fig. 3*A*). Based on these patterns, lateral cortical regions associated with speech and narrative comprehension were chosen for further analysis (Fig. 3*B*; see SI Fig. S3 for corresponding right-hemisphere waveforms; Glasser et al., 2016; Simony et al., 2016; Ji et al., 2019; Fedorenko et al., 2024).

**Figure 3.**
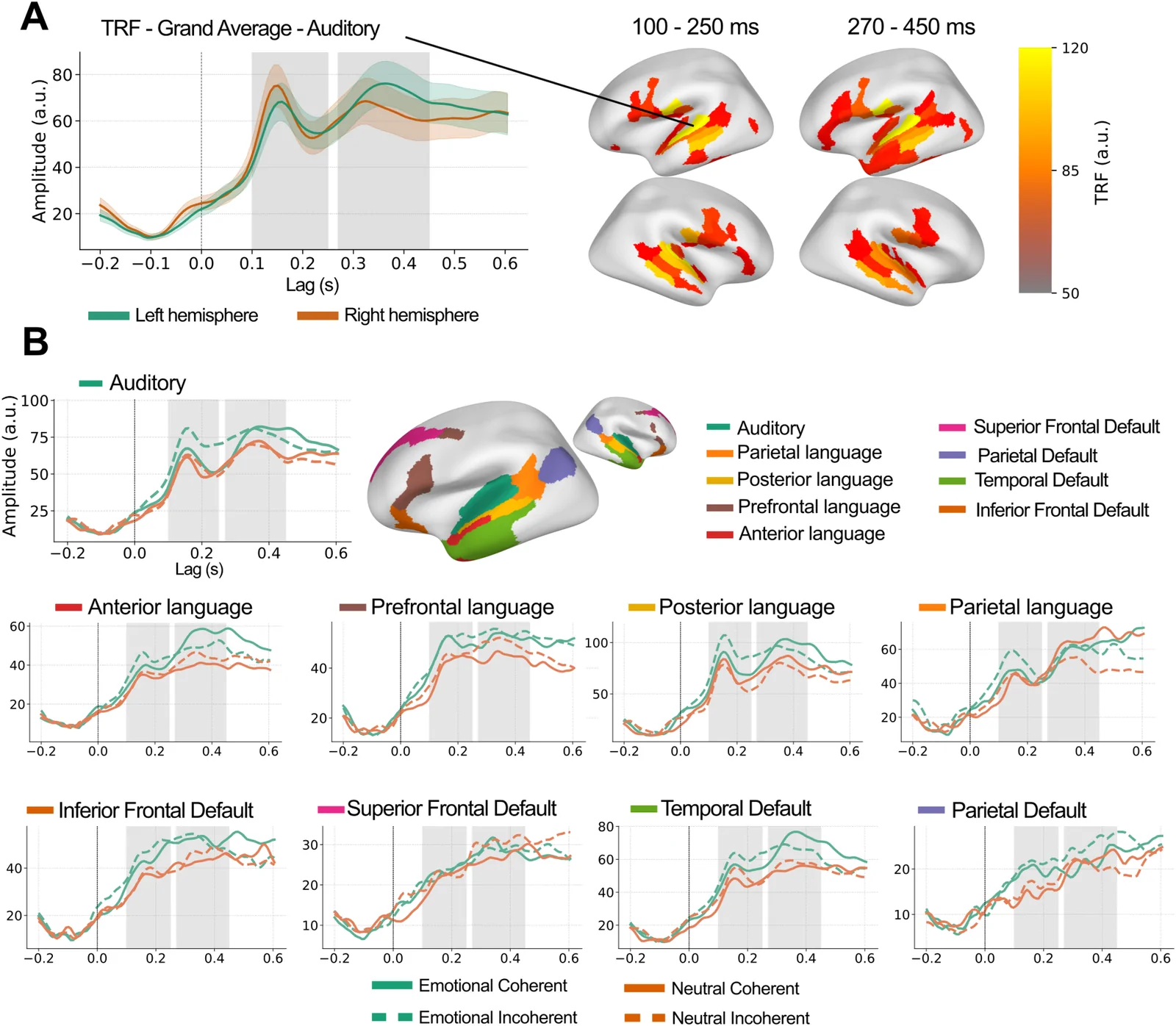
TRFs reveal latency- and region-dependent cortical responses to continuous speech. ***A***, Grand-averaged TRF waveform in the auditory cortex for both hemispheres across all conditions. The gray highlights denote the midlatency (100–250 ms) and late-latency (270–450 ms) windows from which mean activations were extracted for statistical analysis. The parcel-level topographies of TRF responses within the chosen time windows are depicted on the right. ***B***, ROIs chosen for statistical analysis, and left hemisphere TRF waveforms for all conditions in the target dialogues.

In the midlatency TRF window (100–250 ms), the effect of emotional salience on neural responses varied across cortical regions, depending on the dialogue coherence (Emotion × Coherence × ROI: *F*_(4.11,123.21)_ = 4.96, *p* < 0.001; Table 3). Under incoherence, Emotional Salience increased response amplitudes in temporal and parietal regions, while coherent dialogues showed emotional enhancement primarily in the frontal regions (Fig. 4; see SI Fig. S4 for full ROI distributions). Thus, emotional-content modulation of midlatency auditory–language responses depended on dialogue coherence and cortical region.

**Figure 4.**
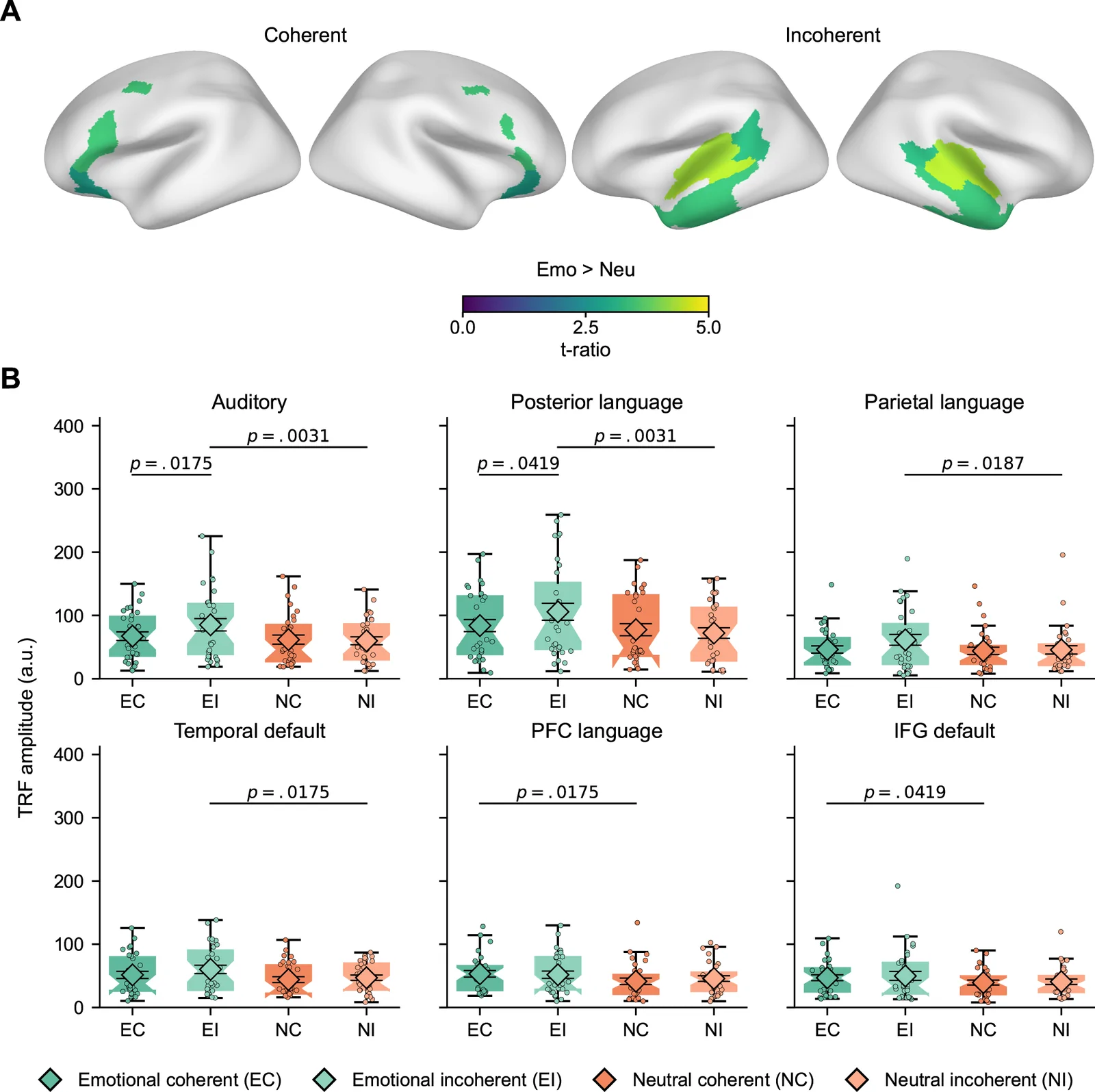
Emotional salience and dialogue coherence modulate midlatency cortical responses. ***A***, Topographical distribution of significant differences between emotional and neutral dialogues in the midlatency window (100–250 ms), shown separately for coherent and incoherent dialogues. Maps show FDR-corrected effects, and color intensity represents the strength of emotional enhancement (Emotional > Neutral) as *t*-ratios. ***B***, Midlatency TRF amplitudes averaged across hemispheres in regions of interest showing significant condition differences for emotional coherent (EC), emotional incoherent (EI), neutral coherent (NC), and neutral incoherent (NI) dialogues. Boxplots and overlaid points show the empirical distributions across participants (*n* = 31). Diamonds indicate estimated marginal means with 95% confidence intervals. Horizontal bars indicate significant FDR-adjusted pairwise contrasts between conditions.

**Table 3.** rm-ANOVA for midlatency TRF amplitudes

Effect	df	*F*	$\eta_{G}^{2}$	*p*
Emotion	1.00, 30.00	17.17	0.014	<0.001
Coherence	1.00, 30.00	3.43	0.003	0.074
ROI	2.16, 64.79	44.02	0.234	<0.001
Hemisphere	1.00, 30.00	1.68	0.002	0.205
Emotion × Coherence	1.00, 30.00	6.22	0.003	0.018
Emotion × ROI	3.50, 105.09	5.73	0.006	<0.001
Coherence × ROI	3.14, 94.24	1.46	0.002	0.229
Emotion × Hemisphere	1.00, 30.00	0.00	0.000	0.949
Coherence × Hemisphere	1.00, 30.00	0.55	0.000	0.463
ROI × Hemisphere	3.27, 98.12	2.67	0.008	0.047
Emotion × Coherence × ROI	4.11, 123.21	4.96	0.004	<0.001
Emotion × Coherence × Hemisphere	1.00, 30.00	1.30	0.000	0.263
Emotion × ROI × Hemisphere	3.52, 105.52	1.23	0.001	0.302
Coherence × ROI × Hemisphere	3.25, 97.54	0.32	0.000	0.826
Emotion × Coherence × ROI × Hemisphere	3.77, 112.98	0.84	0.001	0.497

Type III rm-ANOVA testing the within-participant effects of Emotional salience, Dialogue Coherence, Region of Interest (ROI), and Hemisphere on TRF amplitudes in the midlatency window (100–250 ms). Generalized eta squared $\left(\eta_{G}^{2}\right)$ is reported as the effect size. Exact *p* values are reported unless *p* < 0.001.

In the late-latency window (270−450 ms), emotionally salient speech produced stronger responses than neutral speech (*F*_(1,30)_ = 7.56, *p* = 0.01, $\eta_{G}^{2}=0.007$; Table 4), independent of dialogue coherence (*p* > 0.05). Overall, late TRFs exhibited a broad enhancement for emotional speech in the temporal lobe that was less dependent on dialogue coherence than the midlatency responses (Fig. 5; see SI Fig. S5 for full ROI distributions). Together, these findings indicate that emotional meaning influences attentional tracking of conversations at two stages of speech comprehension: affecting both earlier midlatency auditory–language encoding and later evaluative processing of socioemotional meaning (Schirmer and Kotz, 2006; Kotz and Paulmann, 2011).

**Figure 5.**
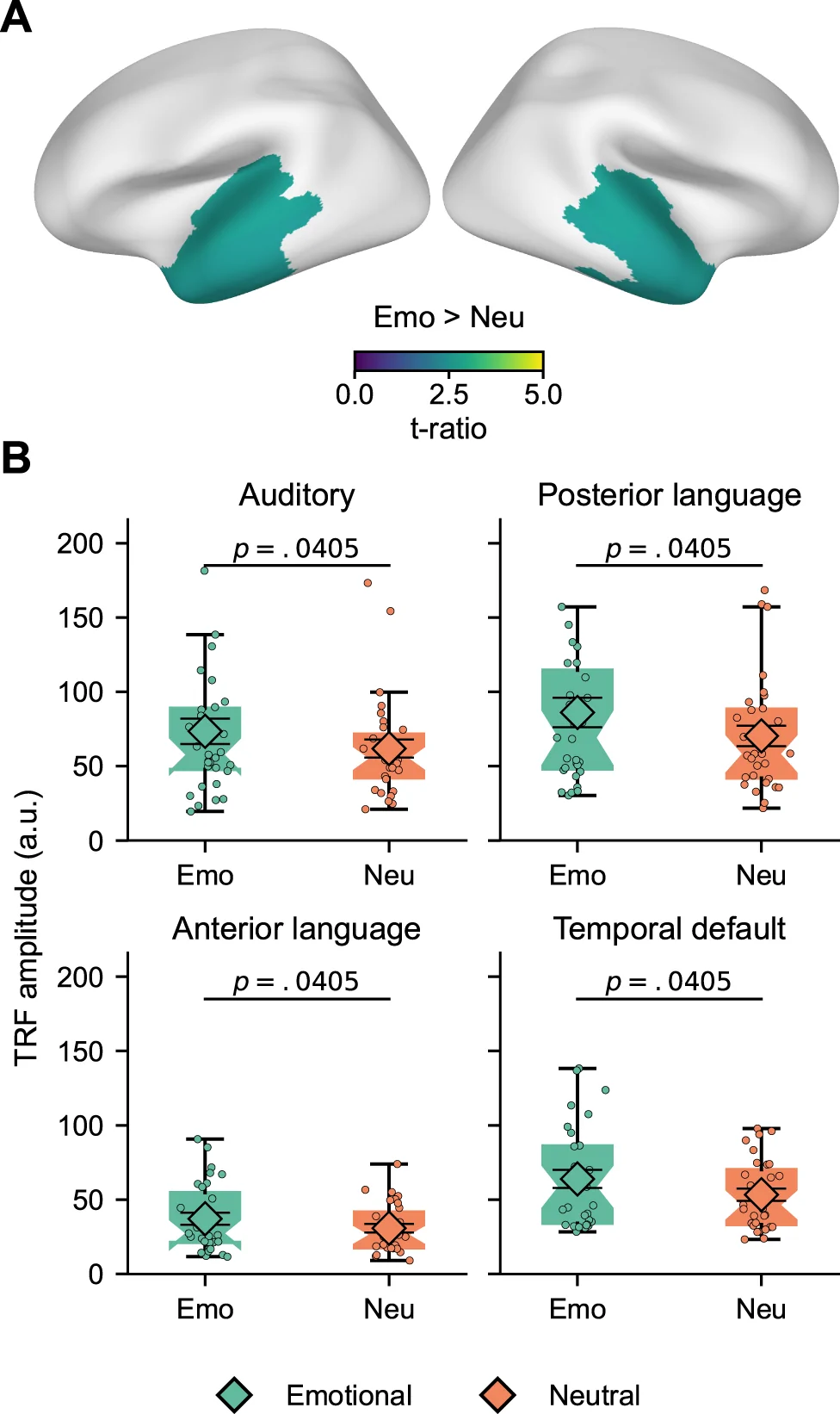
Emotional salience enhances late-latency cortical responses. ***A***, Topographical distribution of significant differences between emotional and neutral dialogues in the late-latency window (270–450 ms), collapsed across dialogue coherence. The map shows FDR-corrected effects, and color intensity represents the strength of emotional enhancement (Emotional > Neutral) as *t*-ratios. ***B***, Late-latency TRF amplitudes averaged across hemispheres in regions of interest showing significant emotional–neutral differences, collapsed across dialogue coherence. Boxplots and overlaid points show the empirical distributions across participants (*n* = 31). Diamonds indicate estimated marginal means with 95% confidence intervals. Horizontal bars indicate significant FDR-adjusted emotional–neutral contrasts within each region of interest.

**Table 4.** rm-ANOVA for late-latency TRF amplitudes

Effect	df	*F*	$\eta_{G}^{2}$	*p*
Emotion	1.00, 30.00	7.56	0.007	0.010
Coherence	1.00, 30.00	1.25	0.001	0.273
ROI	2.37, 71.09	34.91	0.197	<0.001
Hemisphere	1.00, 30.00	9.07	0.017	0.005
Emotion × Coherence	1.00, 30.00	0.51	0.000	0.479
Emotion × ROI	3.04, 91.19	3.60	0.005	0.016
Coherence × ROI	3.22, 96.71	0.48	0.001	0.708
Emotion × Hemisphere	1.00, 30.00	1.62	0.000	0.213
Coherence × Hemisphere	1.00, 30.00	3.45	0.002	0.073
ROI × Hemisphere	3.46, 103.82	2.22	0.008	0.081
Emotion × Coherence × ROI	4.20, 125.87	0.90	0.001	0.468
Emotion × Coherence × Hemisphere	1.00, 30.00	1.25	0.000	0.272
Emotion × ROI × Hemisphere	4.68, 140.25	0.82	0.001	0.530
Coherence × ROI × Hemisphere	3.97, 119.18	2.14	0.002	0.080
Emotion × Coherence × ROI × Hemisphere	2.91, 87.29	1.04	0.001	0.376

Type III rm-ANOVA testing the within-participant effects of Emotional Salience, Dialogue Coherence, Region of Interest (ROI), and Hemisphere on TRF amplitudes in the late-latency window (270–450 ms). Generalized eta squared $\left(\eta_{G}^{2}\right)$ is reported as the effect size. Exact *p* values are reported unless *p* < 0.001.

## Discussion

Using a combined encoding–decoding approach, we show that emotional semantic speech content robustly enhances sustained attentional engagement in conversation in naturalistic cocktail-party settings. By reconstructing the speech envelope from neural activity, we demonstrate that emotional dialogues with negatively valenced, high-arousal and socially significant content enhance neural tracking of attended speech, with a distinct nonlinear temporal trajectory that is shaped by the semantic structure of the unfolding dialogue. Control analyses confirmed that the observed SER effects were not explained by ancillary lexical or semantic differences between the dialogue conditions, such as word typicality, word length, or narrative complexity. Complementary speech-to-brain encoding further revealed context-dependent and context-general effects of emotional salience across frontal, temporal, and parietal brain regions. Together, these findings show that emotional significance conveyed through speech content enhances the neural tracking of attended conversation, while the evolving semantic context shapes the temporal and cortical expression of this enhancement. These effects are observed in a distributed cortical network involved in auditory, semantic, and sociocognitive processing.

Using listeners' brain activity to reconstruct concurrent conversational speech, we found that emotional salience selectively enhanced neural tracking of attended, but not ignored, conversations. While enhanced tracking of attended speech is well established (Ding and Simon, 2012; Zion Golumbic et al., 2013; Fiedler et al., 2019; O’Sullivan et al., 2019), our results extend this work by showing that attentional tracking is further modulated by higher-order socioemotional meaning, particularly when speech conveys negatively valenced, highly arousing, and socially relevant information. The absence of a corresponding emotional-content effect for ignored speech indicates that this enhancement depended on the attended status of the dialogue and is therefore contingent on task relevance. This selectivity was mirrored in participants' subjective ratings, as emotionally salient dialogues were consistently perceived as more affectively intense. Independent validation showed that emotional dialogues combined higher arousal, lower valence, and greater social relevance, suggesting that their emotional significance was carried by a conjoint affective–social profile. Hence, the enhanced neural tracking may not be attributable to negative valence alone (with a possible contribution of negativity bias) but rather to the broader combination of negative valence, heightened arousal, and social relevance. Consistent with this, target SER was related to the combined pattern of arousal, valence, and social relevance. However, this dimensional model did not explain the observed SER better than the categorical emotion contrast, which provided the better overall account.

Complementary TRF analyses showed that this modulation differed across response latencies and cortical regions. At midlatency (100–250 ms), emotionally salient content enhanced responses in auditory, posterior language, and default-mode regions during semantically incoherent dialogues, whereas coherent dialogues showed stronger effects in inferior frontal language and default-mode regions. Thus, emotional-content modulation of midlatency auditory–language responses depended on contextual structure. At later latencies (270–450 ms), emotional content elicited broader enhancements across temporal language and default-mode regions regardless of coherence, suggesting a sustained effect of emotional salience during semantic and sociocognitive integration. Together, these findings indicate that emotional content modulates speech processing at multiple stages through interactions between auditory–language networks and default-mode regions supporting semantic comprehension and social cognition (Mar, 2011; Citron, 2012; Brodbeck et al., 2022; Fedorenko et al., 2024; Yamashita et al., 2025).

SER accuracy for attended dialogues evolved nonlinearly across dialogue turns. Importantly, this temporal profile depended on both the emotional salience of the speech and the dialogue's semantic structure. In emotionally salient coherent dialogues, neural tracking showed a relatively early rise followed by attenuation, whereas emotionally salient incoherent dialogues exhibited a more sustained increase with a later peak. Similar nonlinear changes in attentional tracking of audiovisual speech have been reported previously (Wikman et al., 2021, 2024) and interpreted within an expansion–renormalization framework. In this framework, processing of behaviorally relevant information initially recruits additional neural resources before becoming more selective as an effective task-relevant representation is established (Kilgard, 2012; Wikman et al., 2021, 2024). Importantly, the present findings extend this account by showing that the precise temporal profile varies with both emotional salience and semantic coherence of the content. Emotionally salient sensory events have been associated with attentional prioritization, as they are more likely to offer behaviorally significant information (Astudillo et al., 2018; Schacht and Vrtička, 2018; Fischer et al., 2025). Emotional conversation content may therefore recruit broader processing resources. Consistent with this, emotionally arousing narrative moments have been associated with greater functional integration across large-scale neural networks (Park et al., 2026). Within an expansion–renormalization account and in accordance with our observations, semantic continuity may determine how quickly this enhanced processing stabilizes. In coherent emotional dialogues, successive turns contribute to the same evolving context, potentially allowing enhanced processing to stabilize more rapidly to a more efficient state. In turn, when the content is incoherent, successive turns do not support a stable dialogue-level representation, potentially requiring enhanced processing for longer, which could lead to the more sustained SER increase we observed.

To further examine why semantic coherence may have altered the time course of the emotional enhancement, we turn to predictive-processing theories. Here, our finding that coherence modulated the effect of emotional-content in the midlatency TRFs is of particular relevance. Predictive accounts propose that regularities in ongoing auditory input generate expectations about upcoming information, with violations producing error-related modulation (Winkler et al., 2009; Schröger et al., 2015). Such responses emerge ∼150 ms, where prediction and attention interact in auditory processing, overlapping with our 100–250 ms TRF window (Schröger et al., 2015). Within this window, emotional-content effects were stronger in temporal and parietal regions under incoherence. This pattern is compatible with emotional salience enhancing error-related processing when attended speech conflicts with the evolving context. This increased responsiveness in lower-level auditory processing could then delay stabilization of the enhanced processing state, thereby contributing to the more sustained SER enhancement under incoherence. Because the TRFs did not directly quantify prediction error, this interpretation should be regarded as consistent with, rather than direct evidence of, predictive processing.

Taken together, our findings show that emotionally salient content enhances neural processing of attended conversation, strengthening speech tracking and modulating cortical responses across distributed temporal, parietal, and frontal regions. Importantly, this enhancement was not temporally or contextually uniform, as semantic continuity shaped both how neural tracking of emotional content developed across dialogue turns and how it affected cortical responses. Accordingly, emotional significance may transiently enhance processing across auditory, language, and control systems, while semantic continuity shapes how this enhanced processing evolves across the conversational arc (Clark, 2013; Diederen and Fletcher, 2021; Song et al., 2021; Park et al., 2026). This combination of preferential processing and context-sensitive modulation may be particularly important in real-time interaction, where comprehension and response preparation must proceed in parallel across speaker switches of only 100–200 ms (Stivers et al., 2009; Heldner and Edlund, 2010; Bögels et al., 2015; Brehm and Meyer, 2021; Magyari, 2022). Emotional salience may therefore contribute to the rapid coordination required during emotionally engaging interaction (Stevanovic and Peräkylä, 2015).

### Limitations

In the present study, emotional salience was operationalized as a combination of negative valence, heightened arousal, and high social relevance. This region of the emotional salience space is both empirically grounded and theoretically meaningful, but the study leaves open whether similar effects emerge for other types of emotionally salient conversational content (e.g., positively valenced, high-arousal, socially relevant content). Future research should therefore examine other regions of this space to further delineate the scope of the emotional enhancement effect. Furthermore, emotional expressivity was limited to the semantic content of the dialogues, allowing us to examine how content of speech modulates neural response associated with attentional engagement. However, human expressivity depends on the integration of various expressive modalities, such as paralinguistic features like changes in intonations, as well as communicative gestures like hand movements that likely co-occur with effects presented here. Future work should therefore extend the inquiry to conversations where the participants actively take part in the dialogue and are more free to use their communicative repertoire as a whole.

## Conclusions

Our findings show that emotional salience dynamically shapes attentional engagement in conversational speech in multispeaker environments. Emotional semantic content enhances neural tracking of attended speech and modulates cortical responses to continuous speech. Furthermore, the interaction between emotional significance and semantic continuity demonstrates that cortical responses to socially and motivationally significant cues are shaped by the evolving conversational context. Rather than acting as a simple amplifier of sensory encoding, emotional salience actively recruits and distributes neural resources, reshaping language processing to meet changing conversational demands and enabling flexible adjustment between stable comprehension and adaptive responsiveness.

## Data Availability

The datasets and analysis materials supporting the findings of this study are openly available on the Open Science Framework (https://osf.io/9eyuf/). The repository includes processed data, analysis scripts, and supplementary materials necessary to reproduce the reported results.
